# Fusion of MODIS and Landsat-8 Surface Temperature Images: A New Approach

**DOI:** 10.1371/journal.pone.0117755

**Published:** 2015-03-02

**Authors:** Khaled Hazaymeh, Quazi K. Hassan

**Affiliations:** Department of Geomatics Engineering, Schulich School of Engineering, University of Calgary, 2500 University Dr. NW, Calgary, Alberta, Canada; National Institute of Agricultural Research, FRANCE

## Abstract

Here, our objective was to develop a spatio-temporal image fusion model (STI-FM) for enhancing temporal resolution of Landsat-8 land surface temperature (LST) images by fusing LST images acquired by the Moderate Resolution Imaging Spectroradiometer (MODIS); and implement the developed algorithm over a heterogeneous semi-arid study area in Jordan, Middle East. The STI-FM technique consisted of two major components: (i) establishing a linear relationship between two consecutive MODIS 8-day composite LST images acquired at time 1 and time 2; and (ii) utilizing the above mentioned relationship as a function of a Landsat-8 LST image acquired at time 1 in order to predict a synthetic Landsat-8 LST image at time 2. It revealed that strong linear relationships (i.e., *r^2^*, slopes, and intercepts were in the range 0.93–0.94, 0.94–0.99; and 2.97–20.07) existed between the two consecutive MODIS LST images. We evaluated the synthetic LST images qualitatively and found high visual agreements with the actual Landsat-8 LST images. In addition, we conducted quantitative evaluations of these synthetic images; and found strong agreements with the actual Landsat-8 LST images. For example, *r^2^*, root mean square error (*RMSE*), and absolute average difference (*AAD*)-values were in the ranges 084–0.90, 0.061–0.080, and 0.003–0.004, respectively.

## Introduction

Land surface temperature (LST) is one of the critical biophysical and/or climatic variables that plays an important role in understanding various environmental phenomena [1], such as surface wetness conditions [2], evapotranspiration [3], urban heat island [4], vegetation health [5], forest fire danger conditions [6], vegetation phenology [7], agricultural drought and production [8], and impact of heat on human health [9], etc. Usually, LST is calculated from emitted/outgoing long-wave radiation in the range 8–14 μm measured by either ground or satellite-based instruments/sensors. In general, the ground-based instruments provide accurate estimates at point locations, which are not influenced by the atmospheric conditions. On the contrary, the satellite-based sensors provide an average estimate of LST over each of the grid with a predefined cell size of 1 km x 1 km for Moderate Resolution Imaging Spectroradiometer (MODIS) over large geographic extent of 1100 km x 1100 km where these measurements are highly influenced by atmospheric conditions. Between the two methods, the satellite-based one is very much preferable if the understanding of the spatial distribution of the LSTs is required for an environment issue of interest [10].

At the present time, several LST acquiring satellites are operational, such as MODIS, Advanced Spaceborne Thermal Emission and Reflection (ASTER), Landsat-7 ETM+, Landsat-8 TIRS, Geostationary Operational Environmental Satellite (GOES), NOAA Advanced Very High Resolution Radiometer (AVHRR), Indian National Satellite System (INSAT), Geostationary Meteorological Satellite (GMS), and Meteorology Satellite (Meteosat), etc. One of the interesting features is that these satellites differ from each other in terms of their spatial and temporal resolutions. In general, if a satellite has high spatial resolution, then its temporal resolution is low or vice versa [11]. For example, Landsat-8 and MODIS provide LSTs at 100 m spatial resolution with 16 day temporal resolution and 1000 m with daily time scale, respectively. However, satellite images having both high spatial and temporal resolutions are highly desirable for monitoring environmental issues like agricultural drought, water management, and vegetation phenology [12–15]; which is still unavailable [11]. In order to address this, several spatio-temporal image fusion models were developed during the recent years and primarily focused on producing high spatial and temporal surface reflectance images [16–21]. Note that a limited number of studies were conducted in the area of LST fusion and summarized as follows:

- Liu and Weng [22] adopted a spatial and temporal adaptive reflectance fusion model (STARFM; developed by [16]) to enhance the temporal resolution of ASTER LST images using MODIS for assessing the environmental conditions of west Nile virus over Los Angeles. Note that the STARFM was originally developed to predict synthetic surface reflectance images by fusing Landsat and MODIS images; and comprised of three major steps. Those were the: (i) selection of spectrally similar pixels within a window of interest using Landsat images; (ii) determination of a weighting function factor as a function of both Landsat and MODIS images; and (iii) generation of synthetic Landsat images at time 2 [*synth*-*L(t*_*2*_)] by multiplying the weighting factor defined in step *(ii)* with the sum of difference between two consecutive MODIS images taken at time 1 and time 2 [*M(t*_*2*_)—*M(t*_*1*_)] and Landsat image taken at time1 [*L(t*_*1*_)]. The implementation of the STARFM for LST revealed two issues. For example, it would be impractical over heterogeneous land cover because the surrounding land cover types might have larger impacts on LST characteristics [11]. Also, similar LST-values might be unavailable within the moving window of interest [23–24].
- In addressing the issue of heterogeneous land cover, Weng et al. [11] incorporated annual temperature cycle and linear spectral mixing analysis within the original STARFM model and proposed the spatio-temporal adaptive data fusion algorithm for temperature mapping (SADFAT). They found good agreements between the actual and predicted LST images. However, this method required 3 consecutive MODIS images acquired at time 1, time 2, and time 3 [i.e., *M(t*_*1*_), *M(t*_*2*_), and *M(t*_*3*_)] and 2 Landsat images acquired at 2 times [i.e., *L(t*_*1*_), and *L(t*_*3*_)] for generating the *synth*-*L(t*_*2*_). Thus, it would not be applicable for near real-time applications as the procedure was sort of hindcasting.
- In solving the issue of the selection of similar pixels within a moving window, Huang et al. [23] introduced a bilateral-based method to calculate the predicted value of a pixel using a weighting function of its neighborhood within a moving window instead of using a filter-based method [16]. In another study, Wu et al. [24] provided a variation-based method, which considered the spatial correlations between different pixels within a moving window. However, in both of studies, LST outliers within the moving window of interest affected the predicted images.

In addressing both of the above mentioned issues (i.e., hindcasting and outliers), we developed a data fusion method called spatio-temporal image fusion model (STI-FM) and implemented over heterogeneous semi-arid regions in Jordan, Middle East (see section 3.3 for details). Thus, our objectives were to: (i) generate synthetic Landsat-8 LST images [i.e., *synth*-*L(t*_*2*_)] at time 2 as a function of two consecutive MODIS-based 8-day composite LST images [i.e., *M(t*_*1*_) and *M(t*_*2*_)] acquired at time 1 and time 2 and Landsat-8 LST image [i.e., *L(t*_*1*_)] acquired at time 1; and (ii) validate the outcomes [i.e., *synth*-*L(t*_*2*_)] produced in step *(i)* using the actual landsat-8 LST images [i.e., *L(t*_*2*_)] upon employing statistical methods.

### Study Area and Data Requirements

**Fig 1:**
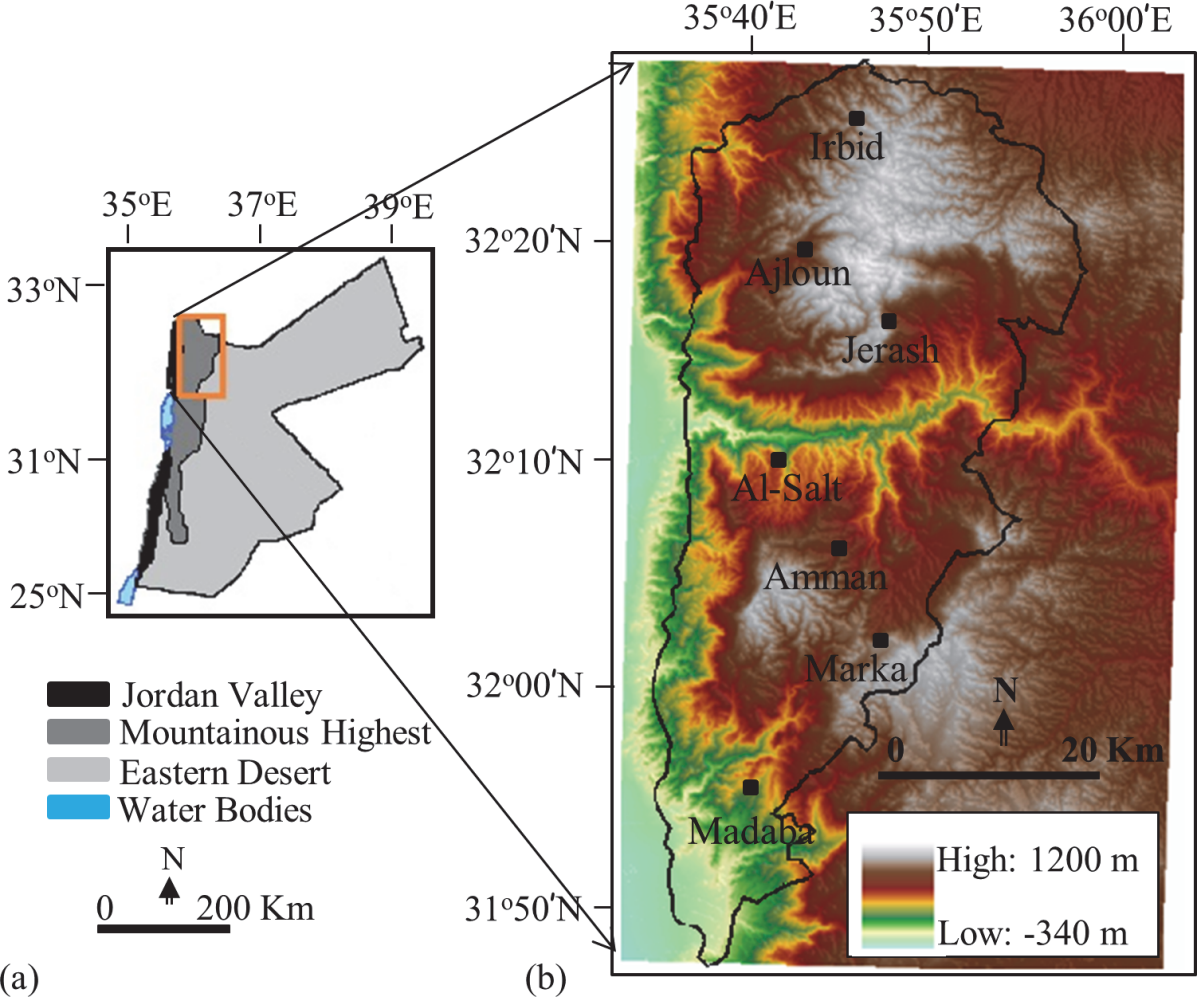
Map of Jordan illustrating three major geographic regions (a); and the study area (as shown using black polygon) over a digital elevation model (b).

Our study area falls in the northwestern part of the mountainous plateau of Jordan, Middle East (Fig. 1A). It is located between 31° 47' to 32° 29' N and 35° 37' to 36° 00' E and covering approximately 3500 km^2^ (see Fig. 1B). The area experiences heterogeneous land cover types, including agricultural lands, such as cereal crops, wheat, barley, lentil, and vetch; orchards such as, olives, apple, nectarine, and vine; barren land; forest; grazing; water body; and urban areas. Topographically, altitudes vary between 600 m and 1200 m above sea mean level, where modest reliefs contain most of the agricultural and populated areas. On the contrary, the steepest reliefs are covered by the forests. The area experiences semi-arid Mediterranean climate conditions with hot and dry summer (∼25°C and no rainfall) during May to August; cool and wet winter (∼7°C and 250–600 mm of rainfall) during November to February; and moderate spring and fall seasons in March/April and September/October respectively. The total annual potential evaporation is ∼ 1900 mm, which primarily observed during the dry season (i.e., May-August) [25].

In this study, we used LST images acquired by Landsat-8 and MODIS satellite systems, which were freely available from U.S. Geological Survey and National Aeronautics and Space Administration (NASA) respectively. Note that both of the data sources are having similar orbital configurations (i.e., 705 km altitude, descending node, sun-synchronous, near-polar, circular, and 10:00 am and 10:30 am local crossing time for Landsat-8 and MODIS, respectively), and spectral properties (i.e., two thermal bands in the ranges 10.60–12.51μm for Landsat and 10.78–12.27μm for MODIS); thus, their thermal properties are consistent and comparable [26–28]. With respect to MODIS dataset, we selected seven 8-day composite LST products/images (i.e., MOD11A2) at 1000 m spatial resolution [29]. The rationale behind the selection of 8-day MODIS composite LST images was to minimize the cloud contamination probability [30]. In case of Landsat-8 LST images, we used four datasets acquired in the spectral range 10.60–11.19 μm at 30 m spatial resolution (which was resampled from 100 m by USGS).The acquisition dates of the employed images are presented in Table 1. Note that we did not use the other thermal band (i.e., 11.50–12.51 μm) of the Landsat-8 due to the large calibration uncertainty (i.e., ∼ RMSE is 1.7 K) associated with it at the time of conducting this study [31].

**Table 1:** The selected MODIS and Landsat-8 images in this study.

MODIS		Landsat-8	
Day of Year (DOY)	Acquisition Dates	Day of Year (DOY)	Acquisition Date
169–176	18–25 June 2013	169	18 June 2013
177–184	26 June—3 July 2013		
185–192	04–11 July 2013	185	4 July 2013
193–200	12–19 July 2013		
201–208	20–27 July 2013	201	20 July 2013
209–216	28 July—4 August		
217–224	5–12 August 2013	217	5 August 2013

## Methods

**Fig 2:**
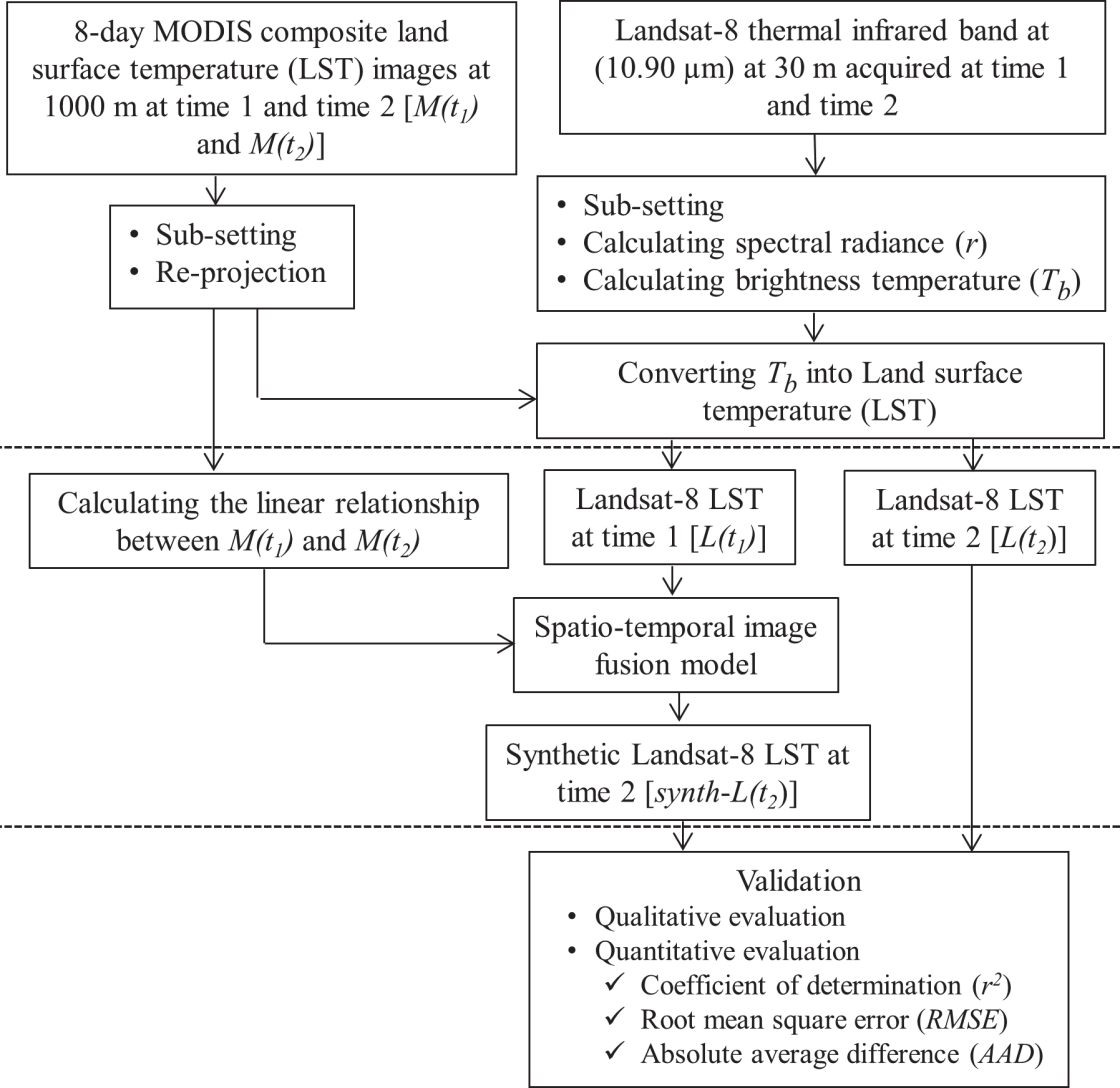
Schematic diagram of the methodology for generating synthetic Landsat-8 LST images at 8 day intervals.

Our methods were divided into four major components and illustrated using a schematic diagram as shown in Fig. 2. Those included: (i) pre-processing of MODIS LST images; (ii) generating Landsat-8 based LST images; (iii) developing a spatio-temporal image fusion model to generate synthetic Landsat-8 LST images at time 2 [i.e., *synth-L(t*_*2*_)]; and (iv) validating the synthetic Landsat-8 LST images by comparing them with actual Landsat-8 LST image. These components are briefly described in the following sections.

### Pre-processing of MODIS LST images

We obtained the MODIS LST images in the Sinusoidal map projection system covering an area of approximately 1100 km x 1100 km. These images were then clipped to the geographic extent of the study area (i.e., Fig. 1B); and re-projected into the Landsat-8 map projection (i.e., UTM Zone 36N with WGS84 datum). Then, we co-registered these images to Landsat-8 images to allow for accurate geographic comparisons and reduce the potential geometric errors (e.g., position and orientation). Finally, we evaluated these LSTs for determining cloud contaminated pixels using the quality control (QC) band (i.e., *1000m QC_Day flags*; another layer available in the MOD11A2 dataset); and excluded them from further analysis.

### Generating Landsat-8 based LST images

The Landsat-8 data was available in the form of digital number (DN). Then, we followed several steps in transforming these DN-values into LSTs. Firstly, we used the Landsat-8 quality assessment (QA) band to determine the cloud contaminated pixels and excluded them from further analysis. Secondly, we converted DN-values into brightness temperature (*T*_*b*_) using the following equations described in [32]:

r=M*DN+A (1)

$$T_{b}=\frac{K_{2}}{\ln(\frac{K_{1}}{r}+1)}$$ (2)

where, *r* is top of atmosphere (*TOA)* spectral radiance in *w/m* ^2^*sr*μm, *M* is band-specific multiplicative rescaling factor, *A* is band-specific additive rescaling factor, *DN* is digital number of the pixel, T_b_ is the at-satellite brightness temperature in Kelvin (K), K_1_ and K_2_ are band-specific thermal conversion constants. The values of *M*, *A*, K_1_, and K_2_ are found in the metadata file of each image.

Thirdly, we transformed the *T*_*b*_-values into LST using MODIS LST images; which was accomplished in three steps. For example: (i) we resampled the spatial resolution of Landsat-8 *T*_*b*_ from 30m to 1000m (i.e., the spatial resolution of MODIS LSTs) by averaging over a moving window of 33x33 pixels; (ii) then we established linear relations between the resampled Landsat-8 *T*_*b*_-values and MODIS LST-values; and (iii) finally, the determined coefficients (i.e., slope and intercept) from the linear relationships in step *(ii)* were used in conjunction with the original Landsat-8 *T*_*b*_ images at 30 m spatial resolution to calculate the LSTs.

### Developing the spatio-temporal image fusion model (STI-FM)

In developing our STI-FM technique, we had two major assumptions. Firstly, there would be linear relationship between the two consecutive MODIS LST images [i.e., *M(t*_*1*_) and *M(t*_*2*_), see equation 3]. This would be the case as temperature regimes usually would follow a distinct temporal pattern if the land cover types wouldn’t change [33]. Secondly, LSTs derived from both Landsat-8 and MODIS images at a particular time period would be similar [e.g., *L(t*_*1*_) ≈ *M(t*_*1*_) or (*L(t*_*2*_) ≈ *M(t*_*2*_)]; because the acquisition of these images were taken place under similar atmospheric conditions [16]. So thus, we determined a linear relationship (i.e., slope *a* and intercept *c*) between *M(t*_*1*_) and *M(t*_*2*_) and then applied with the *L(t*_*1*_) to generate the synthetic Landsat-8 LST image at time 2 [i.e., *synth-L(t*_*2*_)] (see equation 4).

$$M(t_{2})=a*M(t_{1})\mathrm{+c}$$ (3)

$$\mathrm{synth}-L(t_{2})=a*L(t_{1})+c$$ (4)

### Validating the synthetic Landsat-8 LST images

In order to validate the accuracy of the synthetic Landsat-8 LST images, we compared them with actual Landsat-8 LST images in two ways: (i) qualitative evaluation by comparing shapes, textures, and tones of different land cover types in the synthetic and actual images; and (ii) quantitative evaluation using statistical metrics, such as coefficient of determination (*r* ^*2*^), root mean square error (*RMSE*), and absolute average difference (*AAD*) as shown in the following equations:

$$r^{2}={\left[\frac{\sum \left(A_{\left(t\right)}-\bar{A_{\left(t\right)}}\right)\left(S_{\left(t\right)}-\bar{S_{\left(t\right)}}\right)}{\sqrt{\sum {\left(A_{\left(t\right)}-\bar{A_{\left(t\right)}}\right)}^{2}}\sqrt{\sum {\left(S_{\left(t\right)}-\bar{S_{\left(t\right)}}\right)}^{2}}}\right]}^{2}$$ (5)

$$\text{RMSE}=\sqrt{\frac{\sum {\left[S_{\left(t\right)}-A_{\left(t\right)}\right]}^{2}}{n}}$$ (6)

$$\mathrm{AAD}=\frac{1}{n}\sum |S_{\left(t\right)}-A_{\left(t\right)}|$$ (7)

where, *A(t)* and *S(t)* are the actual and the synthetic Landsat-8 surface temperature images; $\bar{A_{\left(t\right)}}$ and $\bar{S_{\left(t\right)}}$ are the mean values of the actual and the synthetic Landsat-8 images; and *n* is the number of observations.

Note that we were able to evaluate the *synth-L(t*_*2*_) images with the actual *L(t*_*2*_) images (i.e., acquired at 16-day intervals); despite the fact that we generated synthetic images at every 8-day intervals.

## Results and Discussion

### Relationship between two MODIS LST images

**Fig 3:**
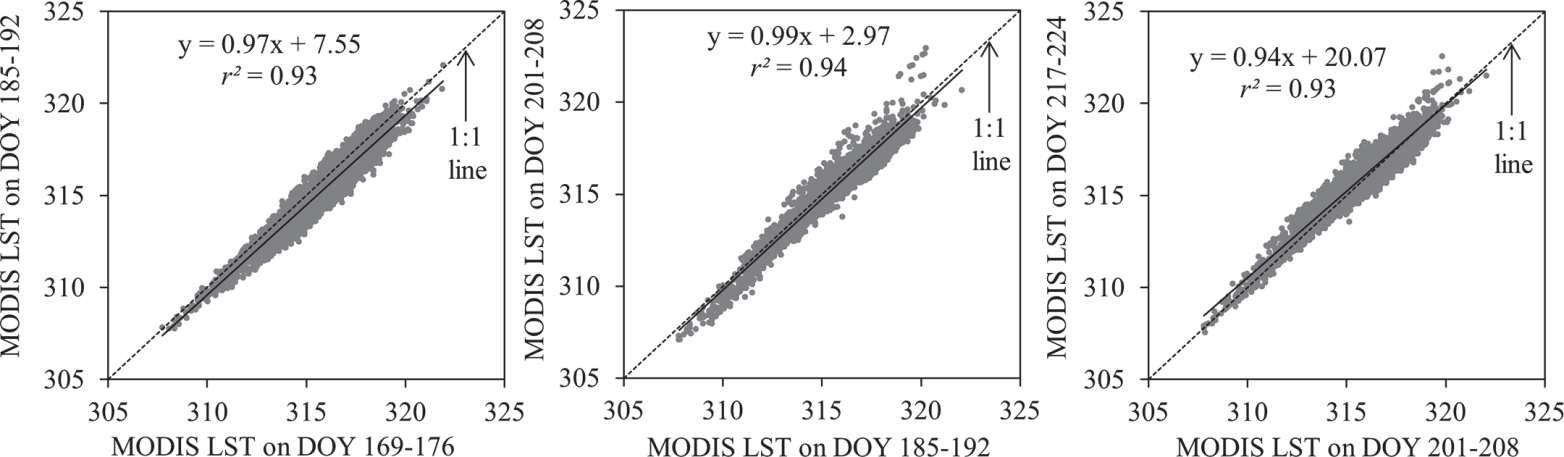
Relation between two consecutive MODIS LST images acquired at time 1 and time 2 [i.e., *M(t*_1_) and *M(t*_2_)] for: (a) DOY 169–176 and DOY 185–192; (b) DOY 185–192 and DOY 201–208; and (c) DOY 201–208 and DOY 217–224.

**Fig 4:**
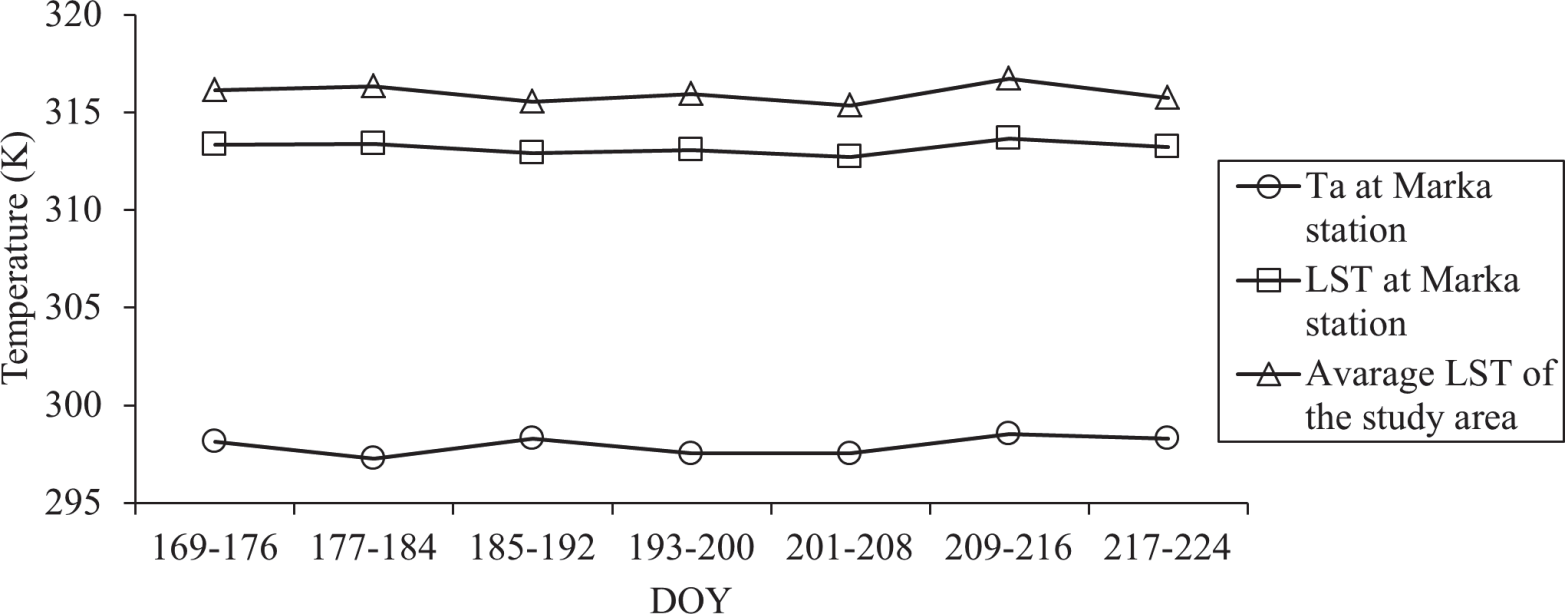
8-day average: (i) air temperature at Marka climate station (see Fig. 1 for the location); (ii) MODIS-derived 8-day LST at Marka station; and (iii) MODIS-derived study area-specific average LST; during the period 18 June 2013 to 24 August 2013.

Fig. 3 shows the relationship between two MODIS LST images, such as DOY 169–176 and DOY 185–192; DOY 185–192 and DOY 201–208; and DOY 201–208 and DOY 217–224. In all cases, we found strong relationships between the LST images, (i.e., *r* ^*2*^ between 0.93–0.94; slopes between 0.94–0.99; and intercepts between 2.97–20.07). Such strong relationships might be explained from the distinct pattern of temperature regimes in the study area (see Fig. 4 for details). Fig. 4 revealed that 8-day average: (i) ground-based air temperature at Marka climate station (see Fig. 1 for the location); (ii) MODIS LST for Marka station; and (iii) MODIS LST for the entire study area were similar for the specific type of measurements/estimates over the period of interest [i.e., DOY 169 (18 June 2013) to DOY 224 (24 August 2013)]. It would be interesting to note that it was not possible to compare our results with other studies as we didn’t find similar ones in the literature so far. Also LST values were found to be higher than ground-based air temperature measurements (see Fig. 4). This would be the case as the surface would be much warmer than the adjacent air masses during summer day-time in mid latitudes (i.e., 25° to 40°) as a result of increased incident solar radiation [34]. It would be worthwhile to mention that these findings supported our first assumption, which thought distinct LST patterns in case of same/similar land cover types.

### Evaluating the synthetic Landsat-8 LST images

**Fig 5:**
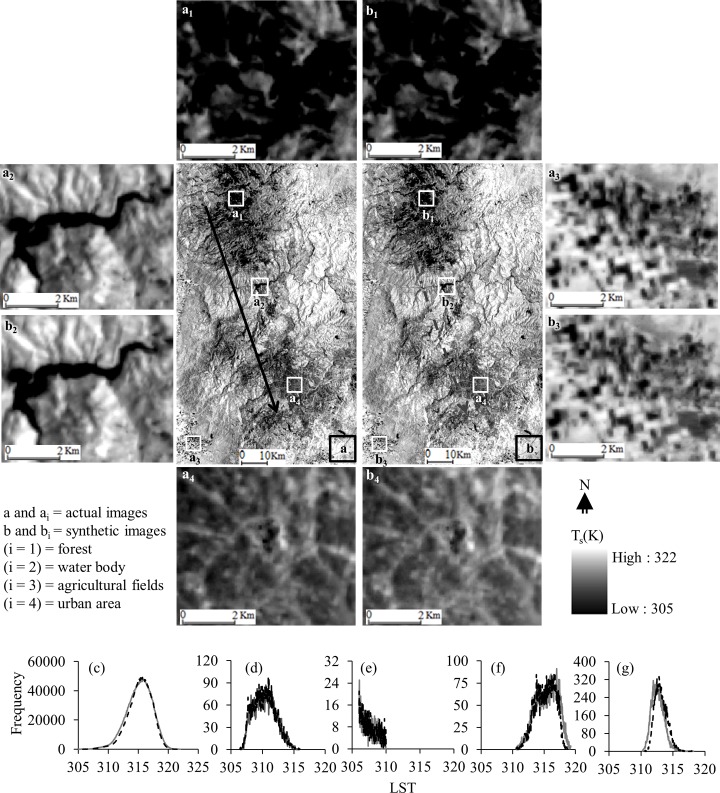
Comparative example between actual (a) and synthetic (b) Landsat-8 LST images for DOY 185 (i.e., 4 July 2013). The panels [(a_1_), (b_1_)], [(a_2_), (b_2_)], [(a_3_), (b_3_)], and [(a_4_), (b_4_)] shows enlarged views over forest, water body, agricultural lands, and urban area respectively for both actual and synthetic images. In addition, the panels (c), (d), (e), (f), and (g) represent histograms of actual (black solid line) and synthetic (gray dashed line) images for the whole study area, forests, water body, agricultural fields, and urban area respectively.

**Fig 6:**
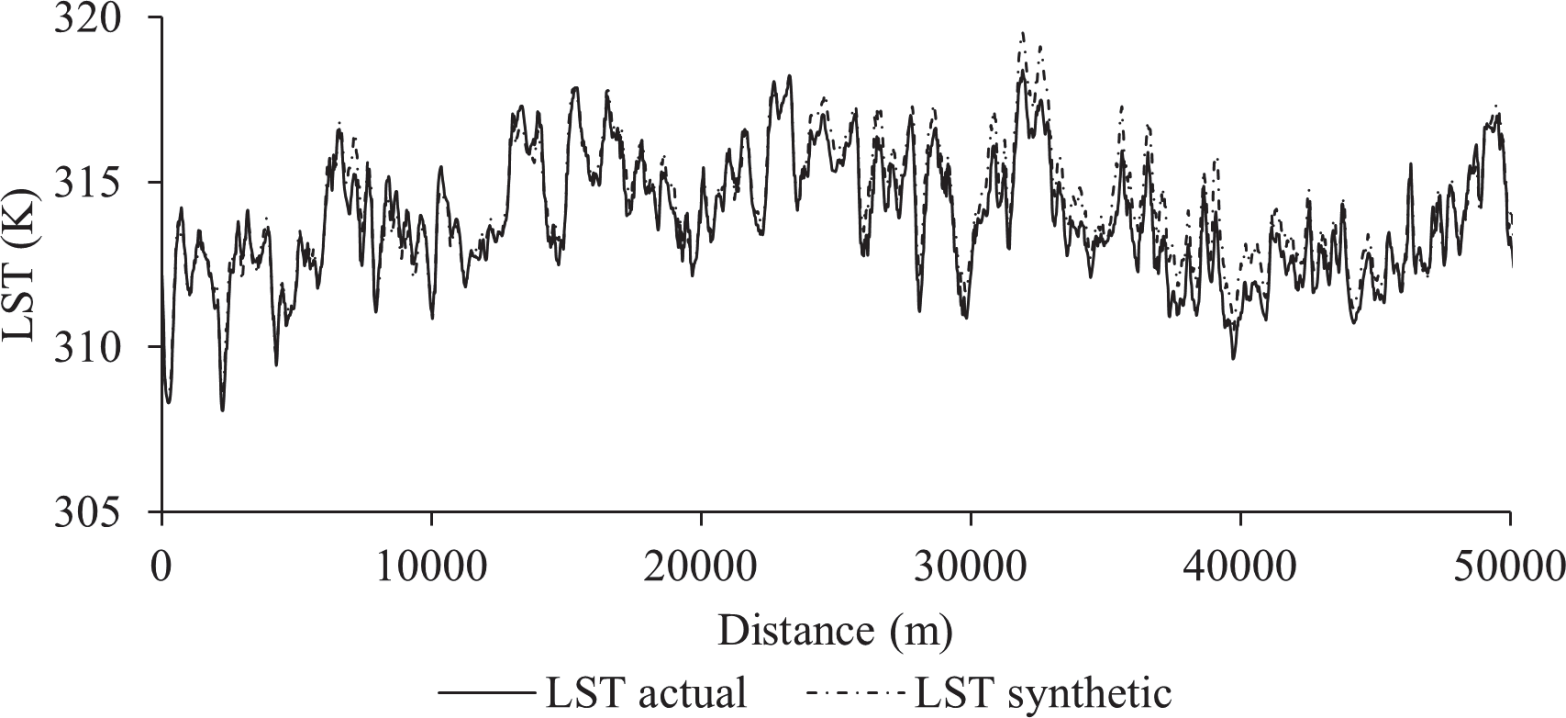
Spatial profiles of the pixels along ∼ 50 km northwest–southeast transect travelling through various land cover types for actual LST image (i.e., the black solid line in Fig. 5A) and synthetic LST image (i.e., the gray dashed line in Fig. 5B).

Fig. 5 shows an example of qualitative evaluation between actual and synthetic Landsat-8 LST images during DOY 185 (i.e., 4 July 2013). This comparison demonstrated an obvious matching in terms of patterns, shapes, sizes, and textures of its features. In this context, we investigated closely four dominant land cover types, such as forests (see Fig. 5 panels a_1_ and b_1_), water body (Fig. 5 panels a_2_ and b_2_), agricultural lands (Fig. 5 panels a_3_ and b_3_), and urban areas (Fig. 5 panels a_4_ and b_4_); and found that the synthetic image predicted LST of these land cover types accurately. In addition to this visual evaluation, we also generated histograms for actual and synthetic LST images for the whole study area and four dominant land cover types; and observed their similarities (see Fig. 5 panels c-g). Also, we extracted the LSTs from both of the actual and synthetic Landsat-8 images during DOY 185 along a transect of about 50 km originated from the northwest to the southeast direction traveling through various land cover types (see the black and gray arrows in Fig. 5). In general, they were similar to each other as the peaks and the spikes of LST values were consistent along the two transects passing through the actual and synthetic LST images (see Fig. 6).

**Fig 7:**
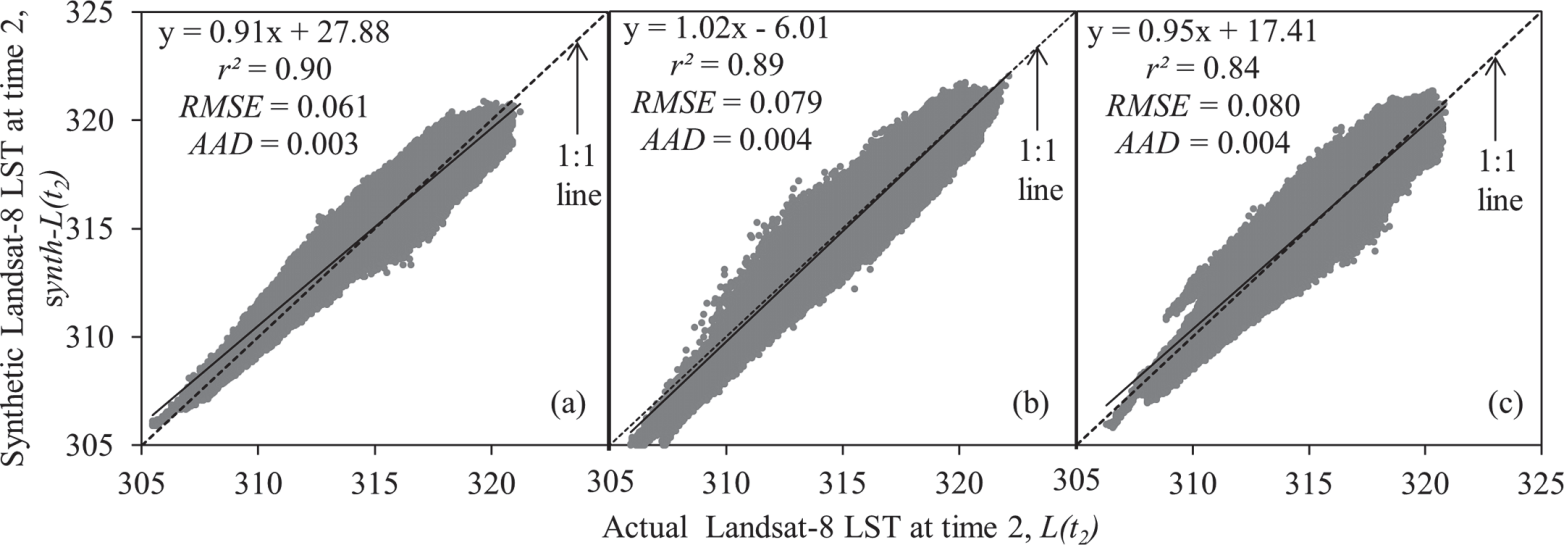
Scatter plots of the relation between actual and synthetic Landsat-8 LST image for: (a) DOY 185 (i.e., 4 July 2013); (b) DOY 201 (i.e., 20 July 2013); and (c) DOY 217 (i.e., 5 August 2013). The dotted and continue lines represent 1:1 and regression line respectively.

In terms of quantitative evaluation, we plotted the actual and synthetic Landsat-8 LST-values for DOY 185, DOY 201, and DOY 217 as shown in Fig. 7. In all of the cases, we found that strong relationships were existed between the variables of interest. For example: *r* ^*2*^, *RMSE*, and *AAD*-values were in the ranges 0.84–0.90, 0.061–0.080 K, and 0.003–0.004 K, respectively. In addition, the regression lines were having close relation with the 1:1 lines, such as the slopes and intercepts were in the ranges 0.91–1.02 and 6.01–27.88 respectively. We also calculated the minimum, maximum, mean, and standard deviation values of each actual and its corresponding synthetic LST images (see Table 2). Note that these values were very close to each other. In fact, the strong agreement between the synthetic and the actual Landsat-8 LST images would support/validate our second assumption that the LSTs derived from Landsat-8 and MODIS sensors would have similar values as they would acquire images almost at the same time under the similar atmospheric conditions.

**Table 2:** Statistical comparisons between actual and synthetic Landsat-8 LST images. The LST values are given in Kelvin.

DOY	LST Image	Minimum	Maximum	Mean	Standard deviation
185	Actual	304.66	321.98	315.21	2.12
	Synthetic	305.69	321.82	315.31	1.99
201	Actual	306.74	320.43	315.21	1.51
	Synthetic	303.75	322.43	315.01	2.30
217	Actual	306.27	321.327	315.44	2.00
	Synthetic	305.08	322.229	315.59	1.44

It would be interesting to note that the results of the model were similar or even better than other studies reported in the literature. For example: (i) Liu and Weng [22] observed less than 0.2 *AAD*-values between the actual and simulated LST images and less than 1°C of standard deviation; (ii) Huang et al. [23] found that the correlation coefficient between the observed and the predicted LST images was in the range 0.72 to 0.83, and *RMSE* was between 0.96 K and 2.6 K; (iii) Wu et al. [24] obtained *AAD* and *RMSE* values of 1.3 K and 1.6 K between the actual and the predicted images respectively; (iv) Weng et al. [11] found good agreements between the actual and predicted LST images with correlation coefficient values in the range 0.87–0.96 and mean difference and *AAD*-values in the range -0.47–1.08 K and 1.25–2.03 K respectively. Though our results showed strong relationships between actual and synthetic Landsat-8 LST images; it would be worthwhile to investigate the following issues:

- In this study, we used MODIS-derived LSTs to calibrate the Landsat-8 *T*_*b*_ at the top of atmosphere in order to generate the Landsat-8 LSTs images. However, this sort of calibration could also be performed using climate data over the study area if available [35].
- Here, we used only one thermal band of Landsat-8 (i.e., 10.60–11.19μm). However, it would be possible to use two available thermal bands (i.e., 10.60–11.19μm and 11.50–12.51 μm) by employing split-window method [36], [37], if properly calibrated second thermal band images would be available [31].
- One of the major requirements of STI-FM would be the use of cloud-free images. However, in some regions or seasons it would be difficult to obtain such images. Therefore, it would be useful to employ gap filling algorithms described in [6] for example to handle the situation.

## Concluding Remarks

In this study, we developed a STI-FM technique and demonstrated its applicability for enhancing the temporal resolution of Landsat-8 LST images from typical 16 days to 8 days as a function of MODIS 8-day composite LST images over a heterogeneous semi-arid study area in Jordan, Middle East. Our results showed strong agreements (i.e., *r* ^*2*^-values in the range 0.84–0.90, *RMSE*-values between 0.061–0.080, and *AAD*-values in the range 0.003–0.004) between the actual and synthetic LST images. We believe that the proposed technique would be applicable for satellite systems that would have similar spectral and orbital configurations other than MODIS and Landsat-8 (e.g., ASTER, MERIS, and AVHRR etc.), and for ecosystems other than semi-arid areas. However, we strongly suggest that the technique should be properly evaluated (that include calibration and validation in particular) prior to adaptation. In addition, we believe that the use of our proposed technique will enhance the temporal resolution of Landsat-8 LST images for different environmental applications; especially those require both high spatial and high temporal information, such as agricultural drought, irrigation management, and crops monitoring.
